# Gelatin-stabilized composites of silver nanoparticles and curcumin: characterization, antibacterial and antioxidant study

**DOI:** 10.1080/14686996.2019.1585131

**Published:** 2019-03-29

**Authors:** Ly Loan Khanh, Nguyen Thanh Truc, Nguyen Tan Dat, Nguyen Thi Phuong Nghi, Vo van Toi, Nguyen Thi Thu Hoai, Tran Ngoc Quyen, Tran Thi Thanh Loan, Nguyen Thi Hiep

**Affiliations:** a Department of Biomedical Engineering, International University, Vietnam National University - Ho Chi Minh City (VNU-HCM), Ho Chi Minh City, Vietnam; b Department of Biotechnology, International University, Vietnam National University - Ho Chi Minh City (VNU-HCM), Ho Chi Minh City, Vietnam; c Institute of Applied Materials Science, Vietnam Academy of Science and Technology, Ho Chi Minh City, Vietnam; d Graduate School of Science and Technology Viet Nam, Vietnam Academy of Science and Technology, Ho Chi Minh City, Vietnam; e Department of Histology, Embryology and Pathology, University of Medicine and Pharmacy, Ho Chi Minh City, Vietnam

**Keywords:** Therapeutic agents, wound treatment, antibacterial, antioxidant, gelatin (Gel), silver nanoparticles (AgNPs), curcumin (Cur), 30 Bio-inspired and biomedical materials, 103 Composites, 211 Scaffold / Tissue engineering / Drug delivery, 503 TEM, STEM, SEM

## Abstract

This is a preliminary study of a material comprising gelatin (Gel), silver nanoparticles (AgNPs) and curcumin (Cur) aimed for wound-healing treatment. Gelatin was used to stabilize AgNPs and encapsulate curcumin to form a therapeutic composite (GelCurAg) for their strong bactericidal and antioxidant properties. GelCurAg formulations with different gelatin concentrations were characterized to attain information about their physiochemical properties and the loading efficiency of therapeutic agents. *In vitro* assessment of GelCurAg focused on antibacterial, antioxidant and cytotoxic aspects. The results suggested that Gel_1_CurAg (synthesized from 1% gelatin solution) could be utilized as potential therapeutic agents in treating infectious wound owing to its bactericidal and antioxidant effects and low toxicity for clinical uses.

## GRAPHICAL ABSTRACT

**Figure:**
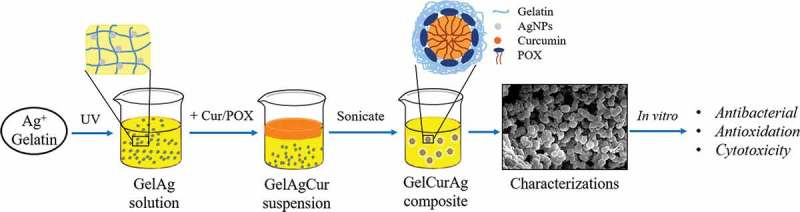

## 1. Introduction

Wound infection has been recognized as one of the main threats in wound treatment [1]. Even when the wound is properly covered by the dressing, bacterial infection can still take place due to the moist environment trapped inside providing microorganism with space and nutrients to grow [1,2]. Therefore, it is crucial to develop wound dressings that can prevent bacteria penetration into the wound or avoid microorganism’ growth [3]. Besides bacterial infection, production of reactive oxygen species (ROS) is another factor needing attention in wound-healing management. During inflammatory phase of normal wound-healing process, immune cells produce ROS to provide defense against microorganism [4,5]. Although low and normal levels of ROS have a positive impact on wound repair and signal transduction for reepithelialization and proliferation of cells, high levels of ROS may disrupt the healing process and induce apoptosis [6].

**Figure 1.**
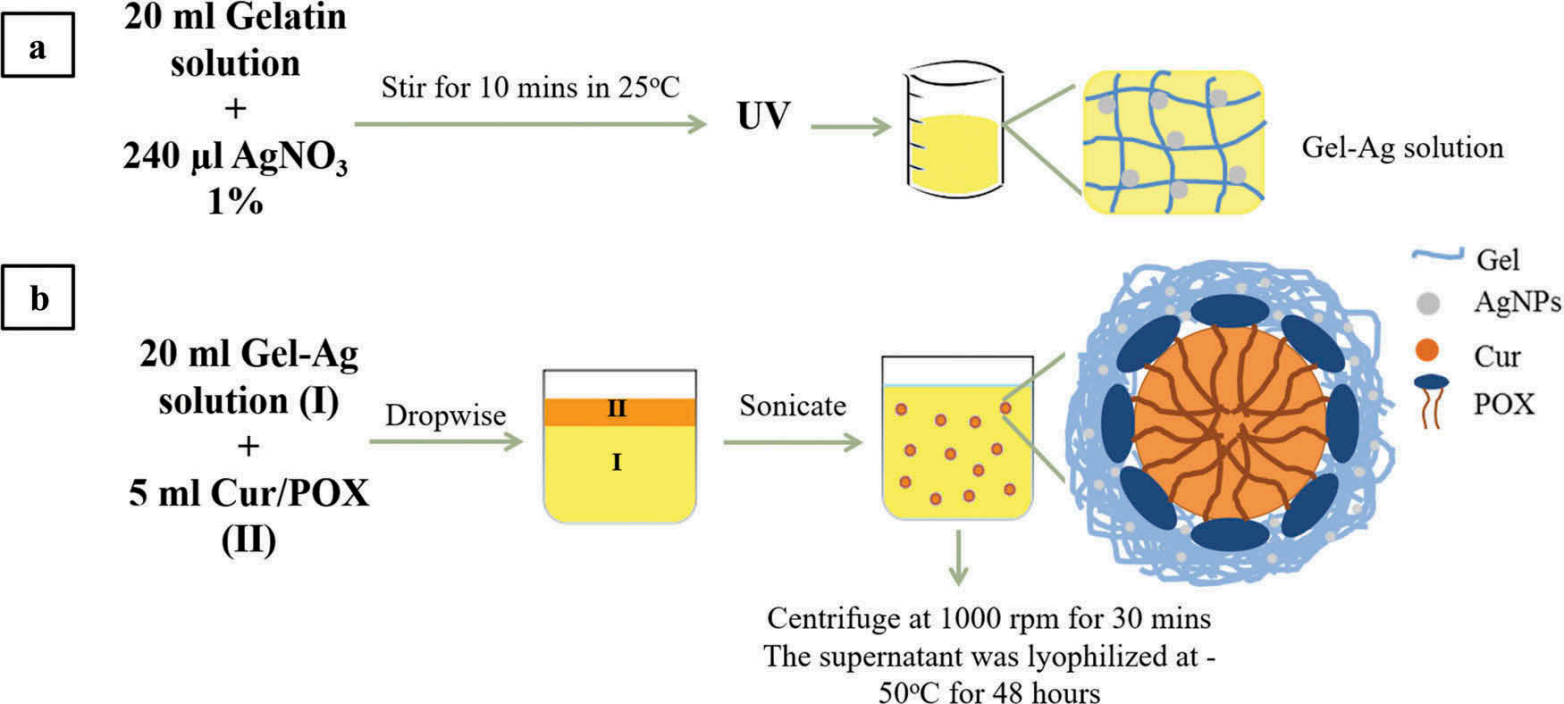
General schematics of methodology: (a) the fabrication of GelAg solution and (b) the encapsulation of curcumin using GelAg solution.

A variety of approaches involving materials with intrinsic bactericidal effect, modified surface or incorporating antimicrobial agents have been used to prepare wound dressings with bactericidal property [3,7]. Among them, the incorporation of nanoparticles (NPs) into wound dressing is a promising alternative to conventional antibiotics. This is owing to the bactericidal activity of NPs against a large number of pathogens, their ability to reduce the unwanted adverse effects of drugs and not trigger microbial resistance [3,6,8]. Silver NPs (AgNPs) have been one of the most prevalent metallic materials that have been utilized in biomedical applications to tackle infectious problems [9,10]. Numerous studies have been conducted to develop AgNPs containing dressings for antibacterial effect including bacterial cellulose hybrid gel-membranes containing AgNPs [11], polycaprolactone (PCL) electrospun membrane containing AgNPs [12] or AgNPs-loaded gelatin in hydrogel pads [13]. Thanh Nhi et al. have successfully employed multi-immersion technique to coat PCL scaffold containing AgNPs embedded in gelatin, which showed sustained antibacterial effect due to the diffusion of AgNPs from gelatin matrix [14].

An approach to control inflammation is to apply curcumin which is a yellow-orange powder extracted from rhizome of *Curcuma longa* (turmeric) and commonly used as a traditional inexpensive herbal medicine [5,15]. Curcumin has been widely used in the topical treatment due to its biological significance of anticancer, antioxidant, anti-inflammatory, antibacterial and wound-healing properties [16–18]. From previous studies, curcumin has been a well-known scavenger of ROS and can reduce the amount of free radicals emerging during inflammatory stage of wound healing [19–21]. Besides, curcumin can also support the growth of fibroblasts, increase blood vessel density and accelerate skin regeneration [21,22]. Most importantly, this compound is deemed to be nontoxic for consumption as up to 8 g/day orally [4].

Despite the potential biological significances, the applications of either AgNPs or curcumin experience inherent drawbacks. Specifically, several researches have revealed that the unique characteristics of NPs (small size, large surface area, chemical composition, solubility and geometry) could also be harmful to human health since they can easily enter human body, cross different biological barriers, reach the most sensitive organs and disrupt cell normal chemical pathways [3,15,16]. Furthermore, the cytotoxicity of AgNPs has been reported for killing mammalian cells at concentrations as low as 2–5 µl/ml [5,10,17]. Meanwhile, the therapeutic efficacy of curcumin is limited due to its low solubility, short half-life and poor bioavailability [17]. Curcumin is also known to be extremely hydrophobic, light-sensitive and practically unstable in alkaline and *in vivo* environment [15,23]. Various curcumin formulations have been developed including emulsified micelles, NPs, nanofibers or hydrogel to overcome those problems [16,24,25]. Among them, the incorporation of curcumin into emulsion-based carrier has emerged as a remedial approach. Gelatin is a natural protein obtained from the hydrolysis of collagen and has been a component in many biomedical applications owning to its biodegradability, biocompatibility and nontoxicity [21]. The diverse functional groups in gelatin structure offers many benefits for chemical modification and covalent drug attachment [26,27]. When drug is encapsulated in gelatin, not only can the drug possess site-specific delivery but also increase drug stability and control drug release effectively [19,27]. In previous research, AgNPs embedded in gelatin (GelAg) solution were successfully synthesized under the effect of ultraviolet (UV) irradiation [12,24]. Aquatic solution containing AgNO_3_ and gelatin under the effect of UV irradiation could produce solvated electrons then reduce the metallic cations to the metallic atoms [23]. Subsequently, AgNPs were stabilized by the amine pendant groups on the gelatin backbone, ultimately leading to the formation of GelAg solution [28]. Previous studies also illustrated the importance of gelatin concentrations on size control and sustained release of AgNPs [29,30], two important factors determining antibacterial activity of AgNPs [31–35]. Therefore, in the current research, gelatin concentration was varied to investigate the range that could provide sufficient stabilizing effect and control release of capping bioactive agents.

In this study, curcumin was incorporated into GelAg emulsion-based carrier using sonoprecipitation method after the preparation of GelAg solution under the effect of UV irradiation. The aim of this is to encapsulate and stabilize therapeutic agents (curcumin and AgNPs) to form a bioactive composite (GelCurAg) for their antibacterial and antioxidant properties implemented in infectious wound treatment. Gelatin concentration was varied from 0.5% to 1.25% while keeping the inputs of therapeutic agents at constant to identify the optimal concentration of gelatin that could provide the sufficient stabilizing effect and control release of encapsulated therapeutic agents. GelCurAg suspensions were characterized to reveal their physicochemical properties using optical absorption, zeta potential measurements, scanning electron microscope (SEM) and X-ray diffraction (XRD). The loading efficiency of AgNPs and curcumin was analyzed using atomic absorption spectroscopy (AAS) and high performance liquid chromatography (HPLC). *In vitro* drug release of curcumin was assessed to predict delivery patterns from GelCurAg suspensions. Last but not the least, the *in vitro* performances of these composites were also studied to evaluate their antibacterial and antioxidant properties and warrant their safety use for clinical applications.

## 2. Materials and methods

### 2.1. Materials

Curcumin powders were supplied by Shanghai Zhanyun Chemical Co. Ltd. Silver nitrate (AgNO_3_ ≥ 99%) and methanol (CH_3_OH) were purchased from Xilong Chemical Co. Ltd. (China). Gelatin (bovine skin, type B), poloxamer 407 (POX 407) and DPPH (1,2- and 2,2-diphenyl-1-picrylhydrazyl) were obtained from Sigma-Aldrich Co. (St. Louis, MO, USA). Mueller-Hinton and tryptic soy media were purchased from Hi-Media (India). Pathogens, including *Staphylococcus aureus* ATCC 25913 and *Pseudomonas aeruginosa* ATCC 9028, were obtained from American Type Culture Collection. A fluorescent lamp was used as the UV light source (FLF-48, 40 W–220 V, 50–60 Hz). All other chemicals used in this study were purchased from major suppliers.

### 2.2. Methods

#### 2.2.1. Fabrication of gelatin-loaded curcumin/silver composite (GelCurAg)

The fabricating process of GelCurAg consists of two stages (Figure 1). First, GelAg solution was synthesized under UV radiation, this solution was then utilized to encapsulate curcumin under the effect of ultrasonication.

(A) Fabrication of gelatin-loaded silver NPs (GelAg) solution

Synthesis of AgNPs using gelatin as green stabilizer was described in the previous report [23]. Briefly, 20 ml of different gelatin solution (0.5%, 0.75%, 1% and 1.25%) was prepared by adding the predetermined amount of gelatin powder (Table 1) in 20 ml distilled water and stirred at the speed of 400 rpm at 40 °C for 1 h. After that, 240 µl of 1% AgNO_3_ solution (equivalent to 120 ppm AgNO_3_) was added to prepared gelatin solutions and stirred at room temperature for 10 min. These mixtures were exposed under UV light for 6 h to form GelAg solutions.

**Table 1.** Formulation compositions (weight basis) for the preparation of GelCurAg.

Code	Gelatin (mg)	Curcumin (mg)	POX 407 (mg)	AgNO_3_ (ppm)
Gel_0.5_CurAg	100	20	250	120
Gel_0.75_CurAg	150	20	250	120
Gel_1_CurAg	200	20	250	120
Gel_1.25_CurAg	250	20	250	120

*The numbers after Gel present the gelatin concentration in wt.%.

(B) Encapsulation of curcumin using gelatin-loaded silver particle (GelAg) solution

The method of encapsulating curcumin followed the sonoprecipitation method [36]. Briefly, 20 mg curcumin and 250 mg POX 407 were dissolved in 5 ml methanol to form solvent phase suspension. This was quickly added dropwise into 20 ml of GelAg solution (anti-solvent phase) while stirring at 750 rpm. The mixture was then sonicated for 20 min using probe sonication (QSONICA, USA) with an amplitude of 30 in an iced-water bath to maintain the temperature below 30 °C. After sonication, the mixture was left stirring for 2 h for methanol to evaporate at 30 °C. In the subsequent step, the mixture was centrifuged at 1000 rpm for 30 min to remove the large particles (pellets), then the supernatant was collected and lyophilized at −50 °C for 48 h.

For the synthesis of Gel_1_Cur as control, 20 ml of 1% gelatin solutions was prepared by adding 200 mg gelatin powder in 20 ml distilled water and stirred with the speed of 400 rpm at 40 °C for 1 h. Then 5 ml methanol containing 20 mg curcumin and 250 mg POX 407 was dropwised quickly to prepared gelatin solution. The subsequent steps followed the same manner as in the synthesis of GelCurAg.

#### 2.2.2. Characterization of gelatin-loaded curcumin/silver composite (GelCurAg)

##### 2.2.2.1. UV*–*visible absorbance

In order to confirm the presence of AgNPs within GelAg solutions and GelCurAg suspensions, multi-plate reader (VarioskanTM LUX multimode microplate reader) was employed for UV–visible absorbance measurement. Briefly, 100 µl of freshly prepared four GelAg solutions, Gel_1_Cur and four GelCurAg suspensions were added into 96-well plate and their optical absorption was measured in the 300–600 nm range.

##### 2.2.2.2. Particle size and zeta potential

The particle size and zeta potential of GelCurAg formulations were analyzed by Zetasizer Nano Series (Malvern Instrument Limited, UK). Particle size was executed with dynamic light scattering at a scattering angle of 173°. Briefly, 10 µl of each GelCurAg suspension was diluted with 10 ml methanol as dispersing solution, followed by vortex shaking for analysis. Then 1 ml of each diluted suspensions was transferred into specialized cuvette for measurement. The measurement was repeated three times.

##### 2.2.2.3. Morphological observation

A scanning electron microscope (SEM, JEOL JSM-IT100, USA) was employed to observe the morphology of four GelCurAg lyophilized samples at 7 and 14 kV. Before SEM observation, the samples were coated with platinum using the coating machine (Cressington Sputter Coater 108 auto, Cressington Scientific Instrument, UK). The average particles size was calculated using ImageJ (Image Processing and Analysis in Java, provide by National Institutes of Health, NIH-USA).

##### 2.2.2.4. Quantitative analysis of silver *NPs*

The loaded amounts of AgNPs in four GelCurAg samples were determined using atomic absorption spectrophotometer (AAS, Shimadzu Corporation, Japan) system. Briefly, 2 ml of sample was treated with 5 ml of concentrated HNO_3_, lightly boiled thoroughly, followed by the addition of 0.5 ml of H_2_O_2_. Then, the mixture was heated and evaporated until only 2 ml of mixture remained. After that, distilled water was added to attain a total volume of 25 ml. After that, AgNPs in each suspension were quantified using AA6650 Flame AAS (Shimadzu system). The sample was diluted to match the standard Ag analysis in HNO_3_ 5% (standard concentration curve ranging from 0.5 to 5 ppm). Triplicate readings were carried out for each sample.

##### 2.2.2.5. Quantitative analysis of curcumin

The quantity of curcumin was determined using Ultimate 3000 HPLC (Thermo scientific Inc., USA). HPLC analysis was carried out using an analytical column (150 × 4.6 mm, 5 µ, C18). For curcumin analysis, the mobile phase consisted of methanol and water in the ratio of 80:20 (v/v) with a flow rate of 1.2 ml/min. The running time and UV–vis detector were set at 5 min and wavelength of 425 nm. A volume of 20 µl of sample was injected into the HPLC system.

*The entrapment efficiency (EE) and drug loading (DL)*: The amount of each GelCurAg formulation was calculated to be equivalent to 1 mg of curcumin corresponding to theoretical mass, the maximum amount of drug (curcumin) could be loaded from the given amount of components as follows. Briefly, the total amount of raw material of each formulation was calculated (the amount of AgNPs was neglected). This total amount was divided by the amount of interest component (20 mg curcumin) to get the amount needed of each GelCurAg formulation. The predetermined amount of each GelCurAg formulation was dispersed in 25 ml distilled water, followed by ultra-sonicating for 30 min at 50 °C to destroy gelatin layer and extract curcumin completely. Then, absolute alcohol (C_2_H_5_OH) was added into the mixture under shaking and cooled down to room temperature and adjusted accurately to obtain 50 ml of total solution. A volume of 100 µl of solution was diluted with 900 µl methanol for HPLC analysis. The measurement was performed three times for each GelCurAg sample and the amount of curcumin entrapped was calculated using the given equations:

$$EE=\;\frac{\begin{array}{c} The\;mass\;of\;drug\;in\;\\ nanoparticles\;x\;100\% \end{array}}{\begin{array}{c} Total\;mass\;of\;drug\;used\;in\;preparation\;\\ \;\;\;\;\;\;\;\;\;\;\;\;\;\;\;\;\;\;\;\;\;\;\;\;\;\;\;of\;nanoparticles \end{array}}$$

$$DL=\frac{The\;mass\;of\;drug\;in\;nanoparticles\;x\;100\%}{The\;mass\;of\;nanoparticles}$$

##### 2.2.2.6. XRD

Crystallographic assay was performed on curcumin powders and GelCurAg samples were acquired by XRD machine (Thermal Nicolet 6700, Cu*K*α, 0.02 deg per step).

#### 2.2.3. *In vitro* studies

##### 2.2.3.1. Drug release

Curcumin release rate of each GelCurAg formulation through dialysis membrane was modified based on previous research [37]. Phosphate-buffered saline containing 20% ethanol was chosen to be the release medium in this study. Briefly, 16 mg of each GelCurAg powders sample was dispersed completely in 2 ml distilled water. These suspensions were introduced into four separate dialysis membrane bags (molecular weight cutoff = 12–14 kDa, Sigma, St. Louis, MO, USA) that were immersed into 60 ml of release medium. The temperature was maintained at 37 °C and the release medium was stirred at 200 rpm. At predetermined time intervals, 1 ml of samples was withdrawn from the medium outside the dialysis bag and replaced by an equal volume of fresh release medium. The concentration of the released curcumin was determined at 425 nm by multi-plate reader (Varioskan™ LUX multimode microplate reader). The percentage of DL of each sample was calculated and displayed graphically. The test was performance in triplicate.

##### 2.2.3.2. Assessment of *in vitro* antibacterial activity

###### 2.2.3.2.1. Microbial culture

The antimicrobial activities of four GelCurAg suspensions were estimated by using minimum inhibitory concentration (MIC), minimum bactericidal concentration (MBC) and agar diffusion methods against two pathogens, Gram-positive (*S. aureus*) and Gram-negative (*P. aeruginosa*). First, pure colonies of each bacterial strains were isolated from preserved strain (stored at −20 °C in glycerol) by using quadrant streak method on Mueller-Hinton agar (MHA) and cultured overnight at 37 °C (the purpose of this method was to separate a mixed culture and to isolate the colonies of each bacterial strain to get a pure culture). Then, a colony was transferred to 10 ml of Mueller-Hinton II broth (MHB II), mixed thoroughly and incubated overnight at 37 °C with rapid shaking. Suspension of each bacterial strain with an optical density at 620 nm OD_620_ = 0.08–0.1 (equal to 0.5 McFarland standards, approximately 1–2 × 10^8^ CFU/ml) was prepared for the further test. For MIC test, 200 µl of bacterial suspension with OD_620_ value of 0.08–0.1 was diluted in 20 ml MHB II culture medium for bacterial seeding. All techniques were conducted under sterile conditions.

###### 2.2.3.2.2. Agar diffusion

The bacterial inhibition of four GelCurAg suspensions was evaluated by agar diffusion method. Briefly, the sterilized MHA solution was poured into Petri dishes. Next, 200 µl of the bacterial suspension was added into and spread out MHA surface. Then, ﬁlter papers disks (about 7 mm in diameter), impregnated with test compounds, were placed on the agar surface. The dishes were incubated at overnight at 37 °C. The zone of inhibition was evaluated by measuring the diameter of the bacterial growth inhibition zone around the membrane (in millimeter). Experiment was conducted in triplicate.

###### 2.2.3.2.3. Determination of MIC and MBC

MIC is the lowest concentration of antimicrobial agents inhibiting the growth of the microorganisms, while MBC is the lowest concentration of an antibacterial agent required to kill a particular pathogen.

Determining the MIC of GelCurAg against two pathogens, *S. aureus* and *P. aeruginosa*, was carried out on 96-well plate. Each plate was dedicated to test one type of bacteria. Four GelCurAg suspensions were tested on the same plate and repeated duplicate. First, MHB II media containing twofold dilution concentration of antimicrobial agents were added to 96-well trays (total volume of media and antibacterial agent was 100 μl). According to original concentration, GelCurAg suspension concentration started from 250 (1st well) to the final dilution of 0.488 µl/ml (10th well). Then, 100 μl of prepared bacterial suspension was seeded into each well from the 1st to the 11th well. Wells at the last 2 rows were used as positive control (11th well) which contained 100 µl bacteria suspension and 100 µl of culture medium and negative control (12th well) which was filled with only 200 μl nutrient broth (used as a baseline). The cultures were incubated for 18–24 h at 37 °C. After that, 10 µl of mixture from the 1st to the 10th well (containing serial reduced concentrations of GelCurAg composites) was inoculated in circle on MHA disks following clock-wise direction while 10 µl of positive control was plated in the middle. The plates were incubated right side up for 18–24 h at 37 °C. The results were read visually with reflected light, observing the presence or absence of macroscopic bacterial growth. The MBC value was determined to be the final point following clock-wise direction at which there was no visible growth of colony while MIC was determined to be the subsequent lower point to MBC in which no or less bacterial growth was observed compared to positive control in the middle.

##### 2.2.3.3. Assessment of *in vitro* antioxidant activity

Antioxidant activities of the encapsulated curcumin were evaluated through a free radical scavenging effect of stable DPPH according to previous research [38]. Brieﬂy, 4 mg of DPPH was dissolved in ethanol which was designated as a pure DPPH solution (0.1 mM). For radical scavenging activity (RSA) determination, pure DPPH solution was added to each sample in 1:3 ratios. Similarly, control was prepared by adding pure DPPH solution to solvent in 1:3 ratios. Finally, the blank solution was prepared by adding the sample to the solvent (ethanol) without DPPH solution in ratio of 1:3. All of the prepared samples were shaken vigorously and incubated for 90 min. The RSA assay was carried out by recording the absorbance of samples containing DPPH at 517 nm at with multi-plate reader (Varioskan™ LUX multimode microplate reader). The following equation was used to calculate the RSAs for each sample:

$$\%\;Inhibition=\left(\;1-\frac{Abs_{sample}-\;Abs_{blank}}{Abs_{control}}\right)*100\%$$

where Abs_sample_, Abs_blank_ and Abs_control_ are the absorbance of sample, blank and control solutions, respectively.

##### 2.2.3.4. Assessment of *in vitro* biocompatibility

The cytotoxicity of the samples was determined using a standard testing protocol-resazurin. Briefly, L929 fibroblast cell cultures are removed from culture flasks by enzymatic digestion (trypsin/EDTA) and the cell suspension is centrifuged (200 *g*, 3 min). The cells are then resuspended in culture medium and counted by hemacytometer. The cell suspension is adjusted at a density of 10^5^ cells/ml. A volume of 100 µl of a cell suspension of 10^5^ cells/ml (10^4^ cells/well) was dispensed into the peripheral wells of a 96-well tissue culture microliter plate. Cells were incubated for 24 h (5% CO_2_, 37 °C, >90% humidity). The plate was examined under a phase contrast microscope to ensure that cell growth is relatively even across the microliter plate. After 24 h incubation, culture medium was aspirated from the cells and replaced with 100 µl of prepared culture medium containing test compounds at concentrations of 250, 125, 62.5, 31.25 and 15.625 µl/ml. The well contained Dulbecco’s Modified Eagle Media (DMEM) medium was used as a control. After 24 h treatment, examine each plate under a phase contrast microscope to identify systematic cell seeding errors and growth characteristics of control and treated cells. Undesirable growth characteristics of control cells can indicate experimental error and can be cause for rejection of the assay. A volume of 10 µl of the 1× resazurin solutions is then added to each test well and the plates are further incubated for 4 h in the incubator at 37 °C. Transfer the plate to a microplate reader (Varioskan™ LUX Multimode Microplate Reader, Thermo Fisher Scientific, USA) to read the fluorescence at *E*_x_/*E*_m_ = 560/590 nm.

#### 2.2.4. Statistical analysis

All experiments were performed in triplicate. The means and standard deviations (SDs) for each set of data were calculated. The results are displayed in form of mean ± SD. One-way ANOVA with Tukey’s *post-hoc* test was used to compare between groups using SigmaPlot 12.5 with the level of significance being 0.05.

## 3. Results

### 3.1. Characterization

#### 3.1.1. Spectroscopic analysis

**Figure 2.**
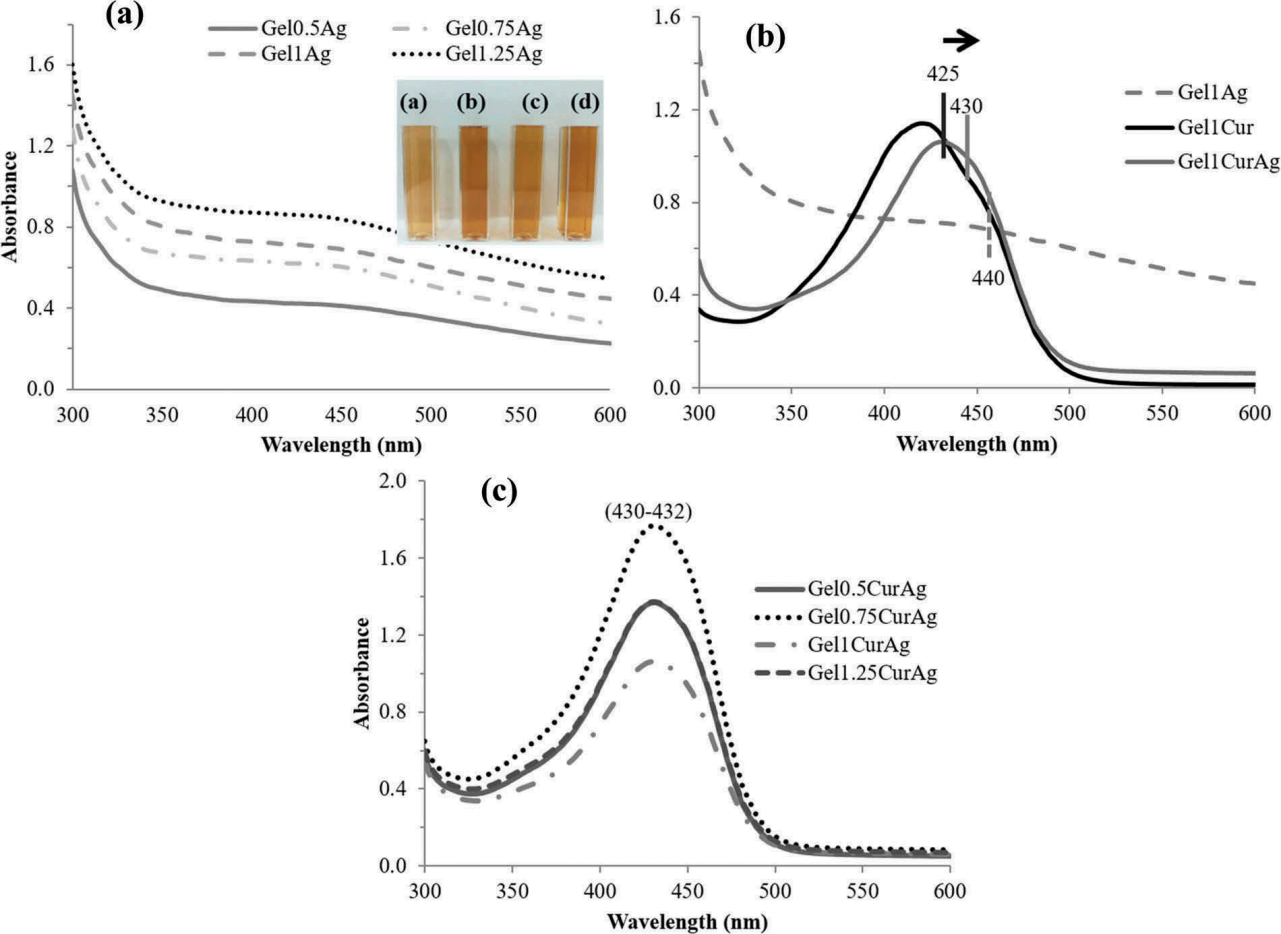
UV–vis absorbance of (a) GelAg solutions: (a) Gel_0.5_Ag, (b) Gel_0.75_Ag, (c) Gel_1_Ag and (d) Gel_1.25_Ag; (b) Gel_1_Cur, Gel_1_Ag and Gel_1_CurAg and (c) Gel_0.5_CurAg, Gel_0.75_CurAg, Gel_1_CurAg and Gel_1.25_CurAg.

UV–vis spectroscopy is one of the most commonly used methods for characterizing the optical response of metal NPs as AgNPs due to their intense surface plasmon resonance (SPR) [3,7]. Figure 2(a) shows optical absorbance of freshly prepared GelAg solutions with different gelatin concentrations. The formation of AgNPs is evidenced by the appearance of GelAgNO3 suspensions, which changed from colorless to yellowish due to the excitation of SPR and the absorption peak appearing at 420–450 nm [23]. Figure 2(b) compares absorbance spectrum of Gel_1_Ag, Gel_1_Cur and Gel_1_CurAg. When AgNPs and curcumin were combined, the absorption peak of Gel_1_CurAg was shifted slightly to the right (430 nm) due to the presence of AgNPs within Gel_1_CurAg. Figure 2(c) shows UV–vis spectra of four GelCurAg suspensions. Since the position of SPR refers to different factors such as size and shape of NPs [39], the similar absorption peak ranged from 430 to 432 nm indicating that most of the GelCurAg suspensions obtained had similar size and shape and gelatin concentration did not alter the size and shape of particles.

#### 3.1.2. Particles size and stability

The parameters in particle size and zeta potential of GelCurAg formulations assessed using Zeta Sizer are displayed in Table 2. Overall, the mean size increased with the increase in gelatin concentration which was not observed through spectroscopic analysis. Furthermore, the particle size distribution was reflected by PdI value, namely the wider range of particles size, the higher PdI values and contrary [40]. The mean PdI values Gel_0.75_CurAg, Gel_1_CurAg and Gel_1.25_CurAg were smaller than 0.4 indicating that these samples possessed even size distribution for monodisperse standards. The stability of GelCurAg suspensions was expressed through zeta potential. The metal NPs with a high absolute zeta potential tend to repel each other and do not form agglomerates while when this figure is low, the particles are aggregated [39]. Thus, it is clearly that Gel_0.75_CurAg and Gel_1_CurAg, having the highest values of zeta, were the most stable formulations.

**Table 2.** Analysis of particle size and stability of GelCurAg composites.

Code	Particle size (nm)	PdI	Zeta potential (mV)
Gel_0.5_CurAg	330 ± 90	0.42 ± 0.15	11.6 ± 1.2
Gel_0.75_CurAg	390 ± 60	0.32 ± 0.03	26.7 ± 1.6
Gel_1_CurAg	375 ± 45	0.24 ± 0.10	27.5 ± 0.9
Gel_1.25_CurAg	730 ± 220	0.38 ± 0.09	11.7 ± 1.0

#### 3.1.3. Morphology observation

**Figure 3.**
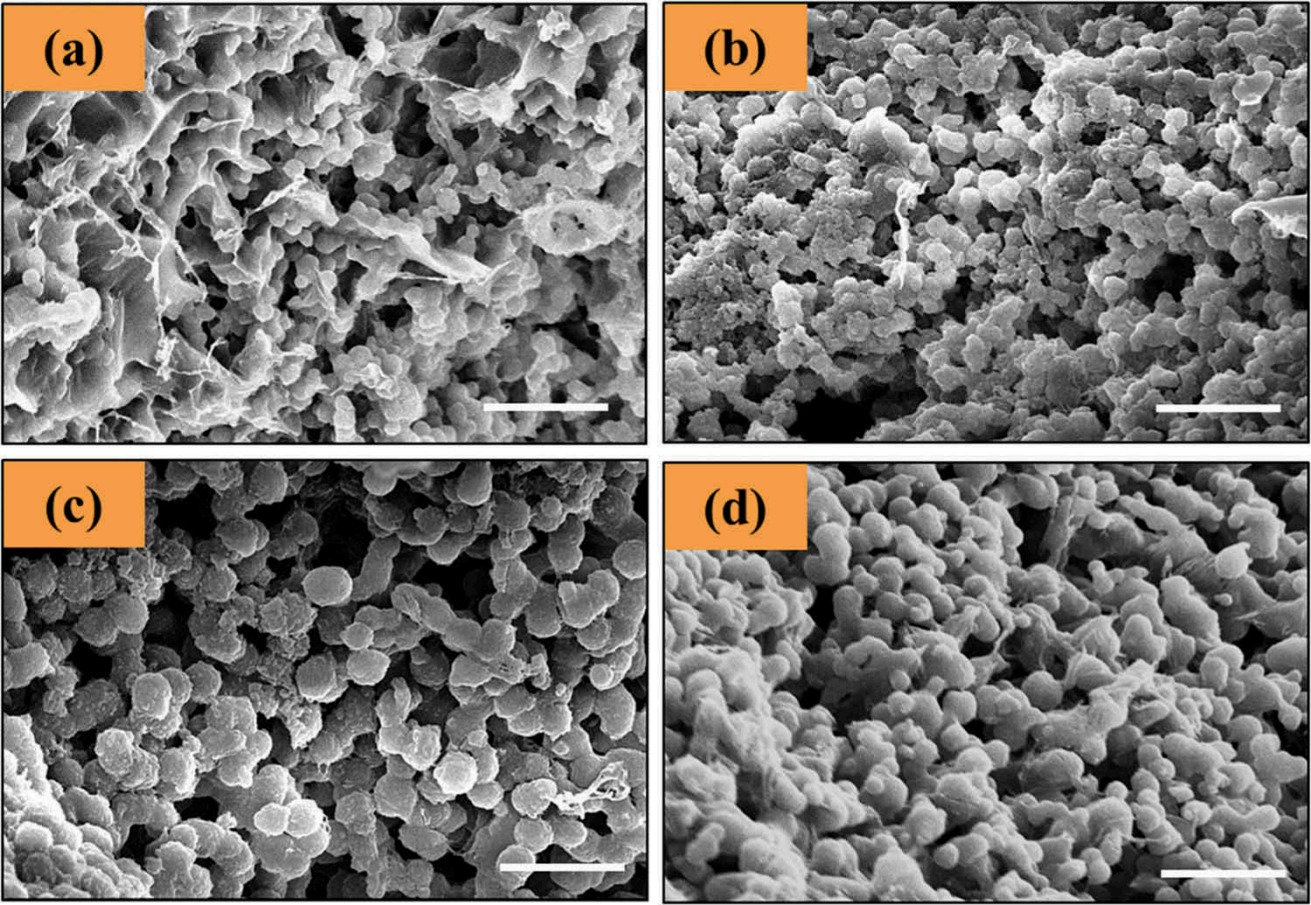
SEM micrographs of (a) Gel_0.5_CurAg, (b) Gel_0.75_CurAg, (c) Gel_1_CurAg and (d) Gel_1.25_CurAg. Scale bar: 5 µm.

Figure 3 shows SEM micrographs of GelCurAg lyophilized samples. It is clearly seen that all formulations produced the freeze-dried spherical particles and the particles size of GelCurAg lyophilized samples rose as increasing the polymer concentration. Specifically, the mean particles size of Gel_0.5_CurAg, Gel_0.75_CurAg, Gel_1_CurAg and Gel_1.25_CurAg was 691, 846, 1156 and 1242 nm, respectively.

#### 3.1.4. Quantitative analysis of silver NPs (AgNPs)

**Figure 4.**
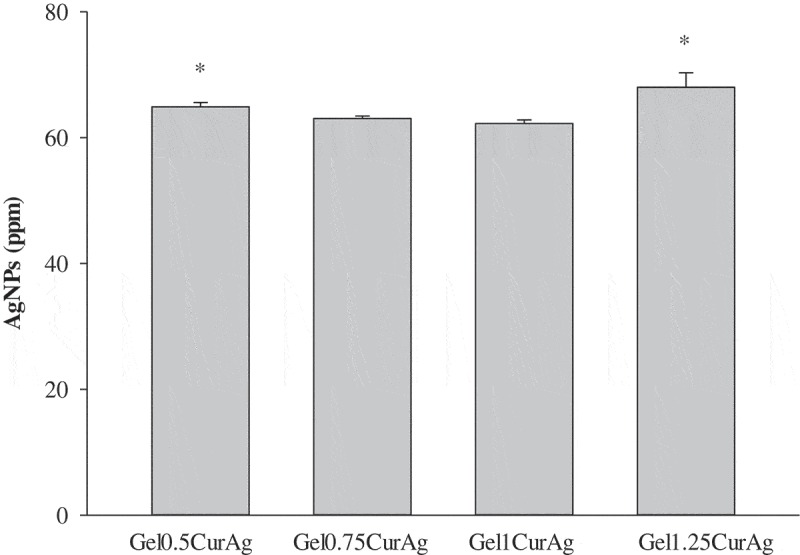
Quantitative analysis of AgNPs within GelCurAg composites. Asterisk (*) indicates *p* < 0.05 (*n* = 3).

In order to obtain precise quantitative analysis of AgNPs, AAS was utilized. Figure 4 shows the concentrations of AgNPs presented within four GelCurAg samples. Overall, the average loading efficiency of these samples stood at around 54.2%. Namely, the corresponding loaded amount of AgNPs within Gel_0.5_CurAg, Gel_0.75_CurAg, Gel_1_CurAg and Gel_1.25_CurAg was 64.9 ± 0.7, 63.0 ± 0.4, 62.2 ± 0.6 and 68 ± 2 ppm, respectively.

#### 3.1.5 Quantitative analysis of curcumin

HPLC was employed to measure the EE and DL of curcumin in GelCurAg lyophilized samples that were given in Table 3. The results show that there were significant differences between EE and DL of four GelCurAg formulations. Specifically, more than 50% curcumin had been encapsulated in different formulations with mean particles size around 364.5 nm (Gel_0.5_CurAg, Gel_0.75_CurAg and Gel_1_CurAg) and the curcumin loading efficiency increased with the increase of gelatin concentration. However, when gelatin concentration reached 1.25%, the amount of curcumin loaded dropped significantly.

**Table 3.** The loading efficiency of curcumin in the studied GelCurAg formulations.

Code	Entrapment efficiency (%)	Drug loading (%)
Gel_0.5_CurAg	52 ± 3*	2.76 ± 0.07*
Gel_0.75_CurAg	55.7 ± 0.4*	2.65 ± 0.02*
Gel_1_CurAg	69 ± 8*	3.0 ± 0.4*
Gel_1.25_CurAg	42 ± 2*	1.61 ± 0.09*

*Indicating the significant difference between samples. The same indicates *p* < 0.05 (*n* = 3).

#### 3.1.6. Crystalline structure

**Figure 5.**
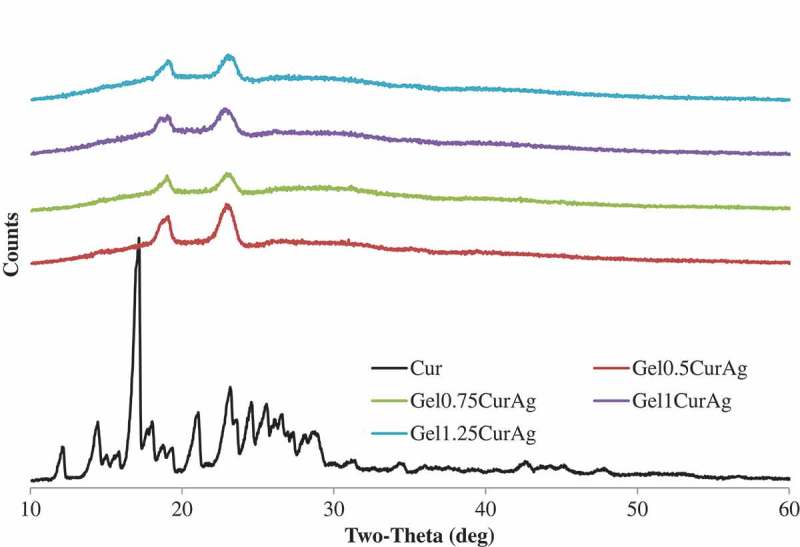
X-ray diffraction patterns of curcumin (black) and GelCurAg lyophilized powders.

The crystallinity of curcumin and four GelCurAg lyophilized powders was assessed using analysis presented in Figure 5. Sharp peaks are absent for curcumin-loaded formulations, suggesting that the curcumin was encapsulated amorphously.

### 3.2. *In vitro* studies

#### 3.2.1. *In vitro* drug release

**Figure 6.**
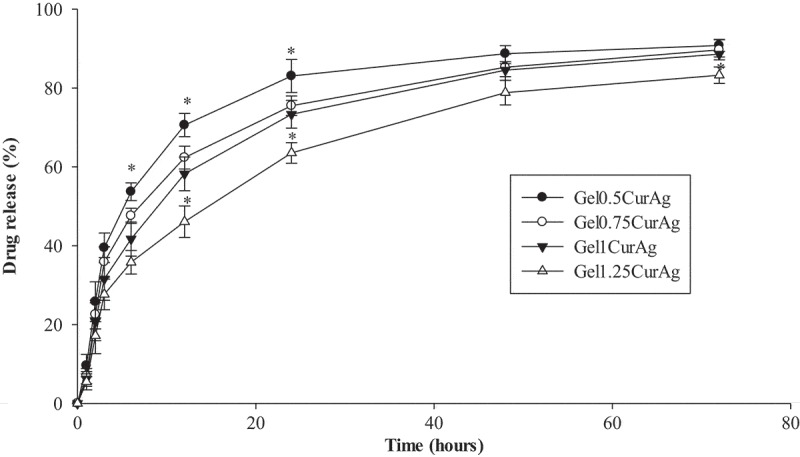
Drug release rate of GelCurAg samples. Asterisk (*) indicates *p* < 0.05 (*n* = 3).

Figure 6 shows the cumulative release profiles of curcumin from four GelCurAg suspensions through dialysis membrane at 37 °C monitored for 3 days. Since curcumin is insoluble in water, PBS containing 20% ethanol was chosen to be the release medium. An initial fast release for about 24 h was followed by a sustained release of drug through dialysis membrane. Particularly, curcumin in Gel_0.5_CurAg suspension released rapidly to over 50% for the first 6 h and gradually rose to around 83% after 1 day. Then, curcumin released sustainably, reaching over 90% after 3 days. The cumulative release of curcumin in other formulations through dialysis membrane shared the same tendency as that of Gel_0.5_CurAg. After 3 days, the curcumin release of other formulations achieved about 83–89%.

#### 3.2.2. Agar diffusion

**Figure 7.**
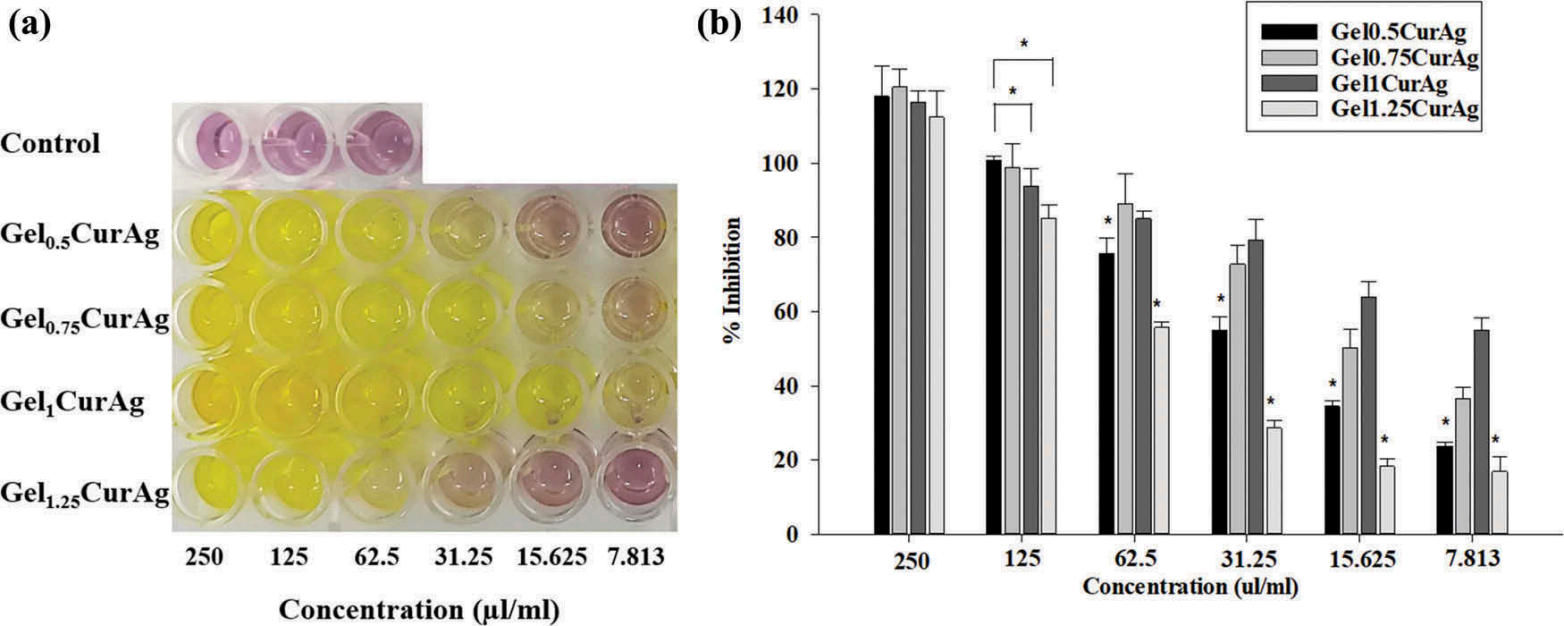
Images of (a) inhibition zone of GelCur and GelCurAg and (b) their measured inhibitory zones against *S. aureus* and *P. aeruginosa.* Asterisk (*) indicates *p* < 0.05 (*n* = 3).

The ability to inhibit the growth of bacteria of four GelCurAg suspensions was investigated using agar diffusion method. The antibacterial activities are presented by inhibition zone; the larger the inhibition zone, the better the bacterial growth inhibition is. Figure 7 presents inhibitory zone of four GelCurAg samples against two strains of bacteria, *P. aeruginosa* and *S. aureus* in comparison with four GelCur control samples. Overall, it can be seen that the antibacterial ability of GelCurAg_12_ relied on the presence of AgNPs since GelCur alone could not inhibit the growth of bacteria (Figure 7(a)) and the lower the gelatin concentration was, the greater the inhibition zone obtained (Figure 7(a,b)). Particularly, GelCurAg revealed antibacterial activity against *S. aureus* for Gel_0.5_CurAg (11 mm), Gel_0.75_CurAg (10.7 ± 0.5 mm), Gel_1_CurAg (10.3 ± 0.6 mm) and Gel_1.25_CurAg (8.5 ± 0.6 mm), respectively, and against *P. aeruginosa* for Gel_0.5_CurAg (10.3 ± 0.4 mm), Gel_0.75_CurAg (9.3 ± 0.5 mm), Gel_1_CurAg (9.0 ± 0.1 mm) and Gel_1.25_CurAg (8.8 ± 0.4 mm).

#### 3.2.3. Determination of MIC and MBC

**Figure 8.**
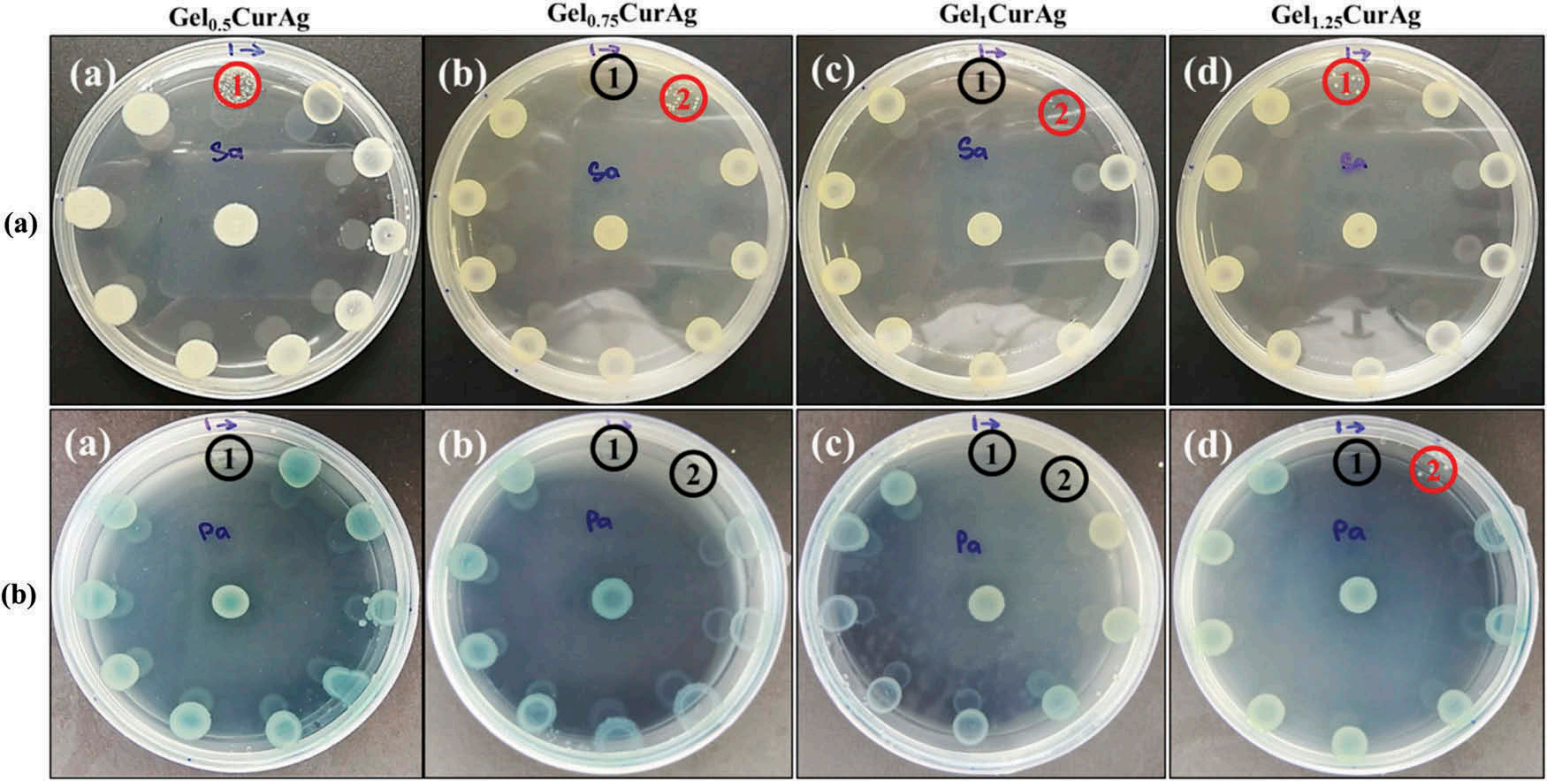
The bactericidal and inhibitory effects against (a) *S. aureus* and (b) *P. aeruginosa* of different concentration of (a) Gel_0.5_CurAg, (b) Gel_0.75_CurAg, (c) Gel_1_CurAg and (d) Gel_1.25_CurAg (MIC [inhibitory] – red; MBC [bactericidal] – black).

Figure 8 shows the results from disk spread method to determine MBC and MIC points of four GelCurAg suspensions against *S. aureus* and *P. aeruginosa*. The MBC value was determined to be the final point following clock-wise direction at which there was no visible growth of colony (black circle). Meanwhile, MIC value might be the subsequent lower point to MBC or right at MBC point, where less or no bacterial growth appeared compared to the positive control in the center. The MIC points are shown by red circled or by black circles if MBC and MIC points are overlapped.

Table 4 summarizes MIC and MBC values of four GelCurAg suspensions against *S. aureus* and *P. aeruginosa*. The prolonged incubation in disk spread method may reduce the antibacterial ability of test samples; therefore, this method was chosen to examine the antibacterial performance of four GelCurAg formulations in extended period of time. At the highest concentration (250 µl/ml), all samples showed the bactericidal effect against *P. aeruginosa*. However, only Gel_0.75_CurAg and Gel_1_CurAg possessed this ability against *S. aureus* at this concentration while Gel_0.5_CurAg and Gel_1.25_CurAg could only inhibit this pathogen growth. At lower concentration (125 µl/ml), Gel_0.75_CurAg and Gel_1_CurAg were able to kill *P. aeruginosa* and inhibit the growth of *S. aureus* while Gel_1.25_CurAg showed the capacity to inhibit *P. aeruginosa* proliferation. When used at 125 µl/ml, the antibacterial properties of Gel_0.5_CurAg did not exhibit through disk spread method. The antibacterial range among four samples stayed in the vicinity of 125–250 µl/ml, which was then chosen to be the focus concentration range in other *in vitro* tests.

**Table 4.** Summary of antibacterial activities of four GelCurAg samples against *S. aureus* and *P. aeruginosa* (unit: µl/ml).

	MIC		MBC	
	*S. aureus*	*P. aeruginosa*	*S. aureus*	*P. aeruginosa*
Gel_0.5_CurAg	250	250	>250	250
Gel_0.75_CurAg	125	125	250	125
Gel_1_CurAg	125	125	250	125
Gel_1.25_CurAg	250	125	>250	250

**Table 5.** Summary of the results of *in vitro* performances of GelCurAg composites.

	Antibacterial					Antioxidant		Biocompatibility	
		MIC (µl/ml)		MBC (µl/ml)					
Formulation	Level of inhibitory zone	*S.a*	*P.a*	*S.a*	*P.a*	250 µl/ml	125 µl/ml	250 µl/ml	125 µl/ml
Gel_0.5_CurAg	1st	250	250	>250	250	✓	✓	✓	✓
Gel_0.75_CurAg	2nd	125	125	250	125	✓	✓	**X**	✓
Gel_1_CurAg	3rd	125	125	250	125	✓	✓	✓	✓
Gel_1.25_CurAg	4th	250	125	>250	250	✓	✓	**X**	✓

*S.a* stands for *S. aureus* and *P.a* for *P. aeruginosa.*

#### 3.2.4. *In vitro* antioxidant activity

**Figure 9.**
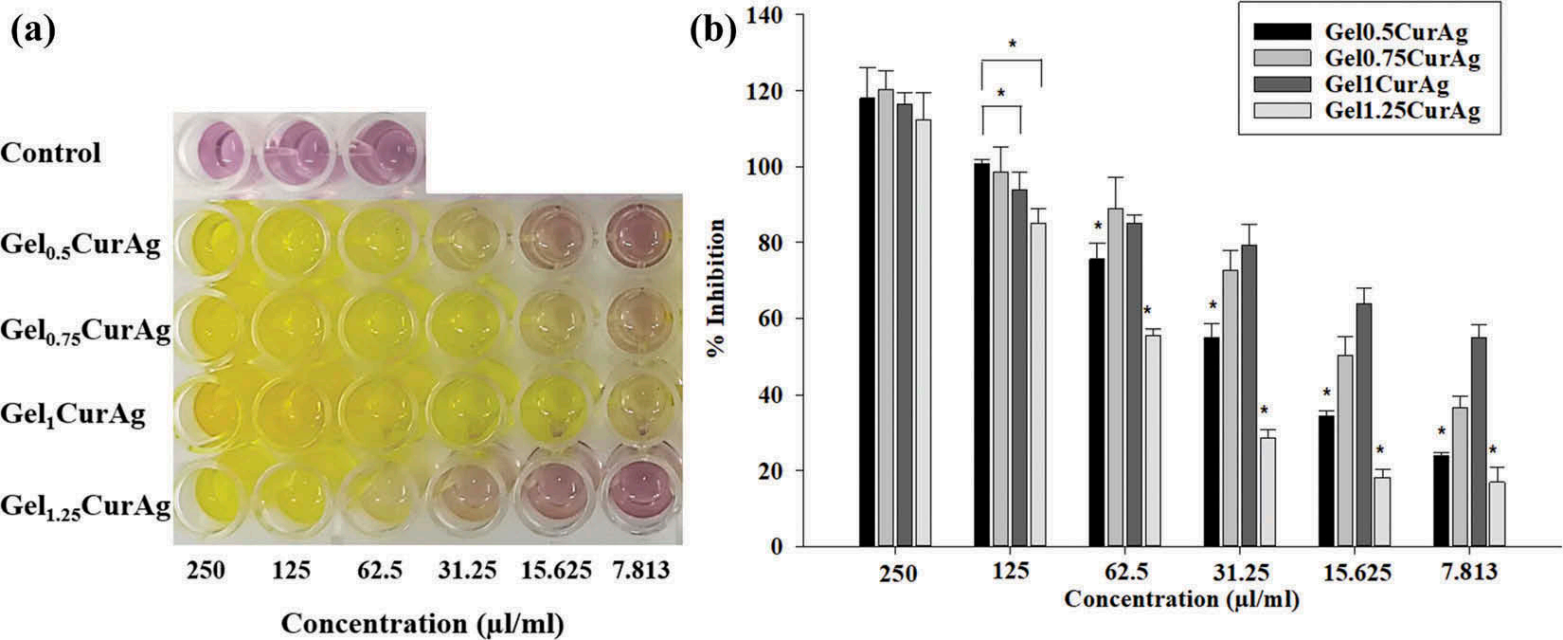
Images of (a) antioxidant activities of different concentration of GelCurAg suspensions through decoloring of DPPH solution and (b) their corresponding percentage of oxidation inhibition after 90 min incubation. Asterisk (*) indicates *p* < 0.05 (*n* = 3).

Figure 9(a) shows the color changes in mixtures containing DPPH and samples at concentrations decreasing from 250 to 7.813 µl/ml. Figure 9(b) reveals that the antioxidant inhibition values among Gel_0.5_CurAg, Gel0_75_CurAg, Gel_1_CurAg and Gel_1.25_CurAg at highest concentration were about 118%, 120%, 116.5% and 112%, respectively. Meanwhile, the data at 125 µl/ml range from 85% to 100%. Thus, in the interest concentration range (125–250 µl/ml), all samples exhibited strong antioxidant activity. When concentration of GelCurAg samples was reduced, the antioxidant also decreased, indicating that this assay was dose-dependent. The results are in agreement with the amount of curcumin loaded in each sample. With lowest curcumin amount, Gel_1.25_CurAg started to lose its ability in inhibiting oxidation at 31.25 µl/ml (28.5%), possessing slightly higher antioxidant ability was Gel_0.5_CurAg, around 34.5% at 15.625 µl/ml. At the lowest concentration (7.8125 µl/ml), Gel_1_CurAg still exhibited for around 50% of inhibition, followed by Gel_0.75_CurAg (36.6%) owning to higher curcumin loading efficiency.

#### 3.2.5. *In vitro* biocompatibility

**Figure 10.**
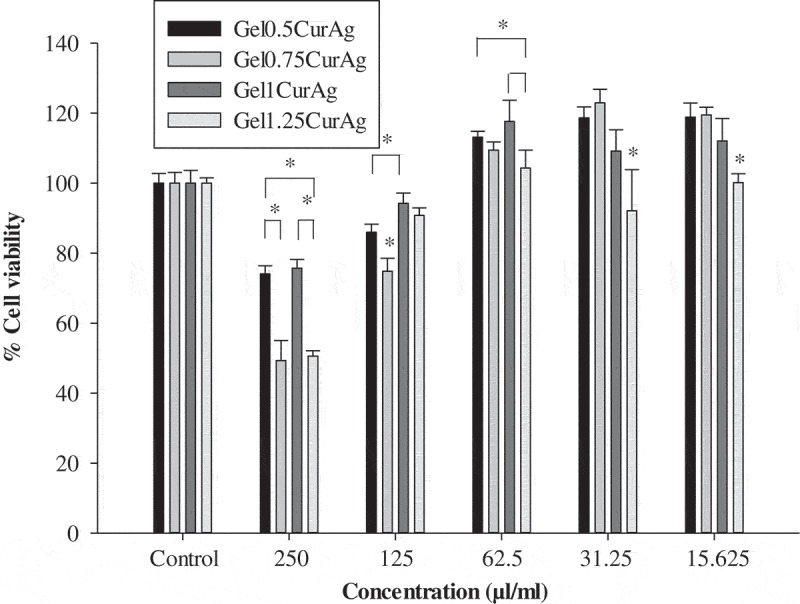
MTT results of different concentration of GelCurAg samples. Asterisk (*) indicates *p* < 0.05 (*n* = 4).

Figure 10 shows the viability of L929 cells after 24 h culture with four GelCurAg composites at different concentrations. This study focuses on the concentration at which GelCurAg formulations can not only inhibit bacterial growth but also remain low toxic to mammalian cells. Particularly, at concentration of 250 µl/ml, Gel_0.5_CurAg, Gel_0.75_CurAg, Gel_1_CurAg and Gel_1.25_CurAg allowed 74.1%, 49.3%, 75.7% and 50.6% cell viability. Meanwhile, there were significant enhancements of viable cells at 125 µl/ml, accounting for corresponding 86%, 74.9%, 94.3% and 90.8%. Based on ISO10993-5, if cell viability at investigated concentration is greater than 70%, materials could be classified as noncytotoxic. So far, two out of four samples met this requirement (Gel_0.5_CurAg and Gel_1_CurAg) when used at 250 µl/ml. Although Gel_0.5_CurAg displayed an outstanding bacterial inhibition through agar diffusion, this formulation failed in maintaining its bioactivities over prolonged period, eliminating this formulation out of potential list for further research. Therefore, only Gel_1_CurAg formulation satisfies the requirements to be potential material for wound-healing treatment.

## 4. Discussion

Whenever body experiences a wound, bacterial infection always happens. In some cases, problems like poor wound care management may lead to severe bacterial infection with continuous invasion of bacteria and formation of biofilm. The invasion of bacteria can be tackled through inflammation. However, biofilm presence contributes to keep the wound in a state of prolonged inflammation by the stimulation of nitric oxide, inflammatory cytokines and free radicals [39,41]. During the inflammatory phase, immune cells produce ROS which provides defense against microorganisms [4,5]. However, high levels of ROS emerging may cause damage to the healing process and induce cell apoptosis, leading to nonhealing state [6]. Therefore, controlling ROS production and providing bactericidal effect are two important factors for successful wound-healing management. Besides, the stabilizing effect and control release of capping agent are important factors in determining performances of therapeutic agents [31–35]. Therefore, gelatin with good stabilizing effect was used to stabilize AgNPs and encapsulate curcumin for their strong bactericidal and antioxidant properties. This composite is hypothesized to adapt to the mentioned requirements for a potential wound-healing material.

First, the formation and presence of AgNPs in GelAg and GelCurAg suspensions were confirmed through spectroscopic analysis. The particles size and shape were determined using zeta sizer and SEM. It is observed that the higher the gelatin concentration, the larger the particles were. This might be due to the increase of viscosity as increasing gelatin concentration was increased, resulting in the growth in the viscous forces that resisted droplet breakdown. Therefore, bigger oil droplets were formed, and subsequently, increasing particles size [42]. Besides, the escalation in particles size observed by SEM could be due to the pressure and stress emerged during lyophilization that altered interactions between particles and caused them aggregate [43].

The loading efficiency of AgNPs and curcumin was measured using AAS and HPLC, respectively. The results showed that the loading efficiency of AgNPs was comparable for all four samples while the loading of curcumin grew with the increase of gelatin concentration. It is known that the bigger the particles, the higher amount of drug encapsulated [6,10]. From previous data in particles size, increasing polymer concentration led to the rise in particle size, thus, increasing the drug content. Besides, the time required for polymer precipitation decreases at higher polymer concentration, so there is less time for drug molecules to diffuse out of particles, therefore, increasing the drug content [44]. Another reason could be due to the availability of a greater amount of gelatin to encapsulate the drug, thus not causing saturation of encapsulation [45]. However, the decreased DL of Gel_1.25_CurAg was observed. Under the effect of centrifuge, large particles carried greater curcumin amount tended to aggregate and were eliminated as pellet, leading to the decrease in DL. This suggested that 1% gelatin was the upper boundary to achieve the highest curcumin loading efficiency. If went over than that, the amount of curcumin loaded would decrease. Despite having been widely used in the topical treatment due to its biological potential of anticancer, antioxidant, anti-inflammatory, antibacterial and stimulating wound-healing properties [11,12,46], low water solubility and hydrolytic instability have limited curcumin applications [17]. The use of emulsion-based formulations for loading and delivery of curcumin is anticipated to enhance its bioavailability and intracellular transport [47]. XRD analysis reveals that curcumin was encapsulated in gelatin layer to form amorphous structure, thereby increasing the solubility of curcumin in aqueous environment.

The *in vitro* release of curcumin mainly depends on three mechanisms: the diffusion of drug through the matrix, the degradation of the polymer and the swelling or dissolution of the polymer [19]. From the release profile it is clear that the cumulative amount of released decreased in accordance with the increasing concentration of gelatin. The decrease in percentage of cumulative drug released could be due to the increased particle size and thus smaller surface area at higher polymer concentration. Besides, the increased polymer concentration could hinder the drug release by diffusion leading to the lower cumulative amount of drug release at higher polymer concentration [45]. Despite having significant effects on stimulating healing process, curcumin may cause toxicity under specific conditions [48]. Therefore, it is essential to determine the percentage of drug release in the initial phase as well as control its release to maintain its bioavailability yet do not cause harm.

Two pathogens, Gram-negative (*P. aeruginosa*) and Gram-positive (*S. aureus*), were purposely utilized for *in vitro* antibacterial assays in this study. Specifically, they are both major pathogens causing community-acquired and nosocomial infections all over the world as well as they also are the most common human wound bacterial isolates regardless of initiating cause of soft tissue injury [44,49]. Last but not the least, infections caused by *S. aureus* and *P. aeruginosa* have been difficult to treat due to their intrinsic ability to resist many classes of antibiotics [12,45]. AgNPs has been widely utilized in biomedical applications to tackle infectious problems owning to its strong bactericidal effects and a broad spectrum antimicrobial activity [50]. It is known that the higher the amount of AgNPs loaded, the greater the antibacterial activities [51]. However, in spite of having the highest quantity of AgNPs, Gel_1.25_CurAg achieved the lowest bacterial growth inhibition. In fact, the inhibition of bacterial growth is strongly associated with the release of antibacterial agents, the greater the AgNPs released, the higher the inhibition zone observed [48,52]. Among all samples, Gel_0.5_CurAg could inhibit bacteria growth the best due to the weakest stabilizing ability, hence leads to the ease of releasing AgNPs into the MHA [53]. Although curcumin only could not inhibit the growth of bacteria, it might still contribute to the overall antibacterial property in the GelCurAg composite. It is studied that curcumin exhibits better activity against Gram-positive bacteria than Gram-negative one and [54], therefore, leads to the enhanced inhibition of bacterial growth of GelCurAg against *S. aureus* than that against *P. aeruginosa* as observed in this study. The determination of MIC and MBC values showed that the antibacterial activity of AgNPs is also related to their size, shape and surface modification by various capping agents, which increases with decreasing particles size [32–35]. Namely, despite having the superior loading of AgNPs, Gel_1.25_CurAg possessed the largest particles size (729 nm) which had lowered its bactericidal ability. Meanwhile, Gel_0.75_CurAg and Gel_1_CurAg with comparable particles size, around 380 nm, displayed the same bactericidal effect. Otherwise, Gel_0.5_CurAg with smaller mean size (329.2 nm) did not show enhanced antibacterial ability as anticipated. This might due to the weak stabilizing and the rapidest release of AgNPs, which then became inactivated over extended period. At highest concentration, Gel_0.5_CurAg only showed the weak inhibition of bacterial growth.

The ability to inhibit oxidation process of different concentrations of GelCurAg samples was also performed using DPPH assay. This assay is based on the capture of the DPPH-free radicals by antioxidants, producing a decrease in absorbance at 517 nm wavelength. DPPH is stable at room temperature and produces a violet solution in organic solvents such as methanol and ethanol, which is reduced in the presence of curcumin, decreasing its coloring, thus the use of DPPH provides an easy, quick path to assess the antioxidant properties of curcumin [55]. The results illustrate that all four GelCurAg formulations were potential to be used as antioxidant agent in wound-healing treatment. Eventually, the biocompatibility has been a great concern for antibacterial material in clinical application. Previous studies have demonstrated the toxic response of AgNPs in different mammalian cell lines [56,57] and curcumin was also known for its ability in triggering some cancer cell lines apoptosis [53,54] causing an urge in conducting analysis to assess the toxicity effect of the manufactured GelCurAg composites on mammalian cells. The cytotoxicity of these composites against L929 cells was investigated using resazurin assay. This assay suggested that Gel_1_CurAg could be a potential biocompatible material in treating infectious wound when used at 250 µl/ml.

Given below is the summary for *in vitro* performances of GelCurAg composites on three aspects: antibacterial, antioxidant and biocompatibility. For antioxidant and biocompatibility, the results were given in form of symbol (✓: pass and X: did not pass).

Despite the potential antibacterial and antioxidant properties, GelCurAg composite hinders some shortcomings toward wound healing. Particularly, one of the important criteria of a wound dressing is to provide and maintain moist environment to the healing wound [59]. However, the wound dressing containing GelCurAg composite having rapid degradation profile in aqueous environment may accelerate the dehydration underneath the dressing, thus disrupting the healing wound as it dries and even damaging the tissue when it is removed [27,59]. Furthermore, the poor mechanical strength of gelatin could be another disadvantage that discourages the composite application in wound healing [60]. While the former drawback could be addressed by using chemical crosslinker, the mechanical properties of the composite could be improved by combining with other polymers [14,75]. It is also interesting to note that fibroblasts survival increased considerably, surpassing even the control sample, when GelCurAg suspensions were applied at 62.5 µl/ml or lower. This suggests that GelCurAg could support cell proliferation and needs to be investigated further.

## 5. Conclusions

In this research, four GelCurAg composites were characterized and evaluated through *in vitro* studies in order to obtain information about physiochemical properties in relation with the *in vitro* performances of GelCurAg composites on three aspects including antibacterial, antioxidant and cytotoxic effects. Agar diffusion results showed that the inhibition of bacteria growth was strongly associated with the release of antibacterial agents from GelCurAg composites and the lower the gelatin concentration, the higher release of those agents and larger inhibition zone observed. Further analysis in determining MIC and MBC values given that the antimicrobial effect of Gel_0.75_CurAg and Gel_1_CurAg was significantly higher than other formulations in GelCurAg group. *In vitro* DPPH assay demonstrates strong antioxidant activity of all four GelCurAg composites when used at 125–250 µl/ml. The results in cytotoxicity suggested that Gel_1_CurAg could be a potential biocompatible material in treating infectious wound at 250 µl/ml owning to its enhanced antibacterial and antioxidant properties.
